# Safety and efficacy of duloxetine treatment in older and younger patients with osteoarthritis knee pain: a post hoc, subgroup analysis of two randomized, placebo-controlled trials

**DOI:** 10.1186/1471-2474-14-137

**Published:** 2013-04-17

**Authors:** Joseph L Micca, Dustin Ruff, Jonna Ahl, Madelaine M Wohlreich

**Affiliations:** 1 Patient Centered Healthcare, Atlanta, GA, USA; 2 Lilly USA, LLC, Drop Code 4133, Indianapolis, IN, 46285, USA

**Keywords:** Duloxetine, Osteoarthritis of the knee, Older patients, Pain

## Abstract

**Background:**

Osteoarthritis (OA) knee pain is common in older patients and contributes to decreased quality of life. Older patients are generally at higher risk of adverse drug reactions due to age-related changes in physiology that affect drug disposition, metabolism, and response. These analyses examined efficacy and safety outcomes of older (≥65 years) versus younger patients from clinical trials of duloxetine in the management of OA knee pain.

**Methods:**

This is a post hoc analysis of two 13-week studies, in which patients were randomized to duloxetine 60 mg/day or placebo. Both studies allowed potential dose changes after 7 weeks of dosing, with Study I re-randomizing duloxetine treated patients to either stay on 60 mg/day or increase to 120 mg/day; while Study II more closely mimicked clinical practice by escalating only non-responding patients to 120 mg/day. For all analyses patients were subgrouped by age: older (≥65 years) and younger (40–64 years). Overall efficacy and safety age-group comparisons of duloxetine versus placebo were performed using pooled data from both studies with all duloxetine dose levels combined. Safety analyses included discontinuation rates, treatment-emergent adverse events, and serious adverse events. To evaluate the effects of increasing the dose in non-responding patients, only Study II data were evaluated. Treatment arms were defined post hoc as placebo, duloxetine 60 mg/day, and duloxetine 60/120 mg/day.

**Results:**

At study end, patients in each age group who were treated with duloxetine versus placebo had significantly greater improvement in pain (both, p<.05), and there was no significant effect of age on treatment (p=.72). Increasing the dose to 120 mg in non-responding patients was not found to have a significant advantage. Among treatment-emergent adverse events with duloxetine treatment, only dizziness had a significantly differential treatment effect (p=.02) with greater incidence over placebo in younger patients (6.6% versus 0.6%, p=.02), but not in older patients (1.0% versus 3.2%, p=.29).

**Conclusions:**

Duloxetine was efficacious and generally well tolerated for management of symptomatic knee OA in both older and younger patients, but increasing the dose to 120 mg in non-responding patients did not provide additional benefit.

## Background

Osteoarthritis (OA) of the knee is the most common form of arthritis in older individuals and a leading cause of pain and disability [1], which contributes to overall decreased quality of life, [2] as well as the increased risk of depression and anxiety [3,4]. Alleviation of pain symptoms is paramount in managing knee OA, and should be the focus of treatment [5]. The approach to pain management in older patients may differ from that for younger patients, due to generally higher risk of adverse drug reactions from age-related changes in physiology that affect drug disposition, metabolism, and response [6]. Since many adverse events are dose related, appropriate dosing for analgesic medications in this sensitive population is a particularly important clinical concern.

Prescription pain management options for OA knee pain include topical or oral non-steroidal anti-inflammatory drugs (NSAIDs), intra-articular injections of corticosteroids and hyaluronates, and opioids [5]. NSAIDs and opioids are associated with potential use-limiting risks or side effects which may be heightened in the older patient [7]. These effects include gastrointestinal bleeding, peptic ulcer disease, nephrotoxicity, and serious long term cardiovascular effects with NSAID use, and risks of sedation, respiratory depression, overdose, misuse, or dependency with opioids [7]. When treating OA knee pain in older patients, it is important to have alternative treatment options that offer efficacy with an acceptable risk profile in this population.

Current evidence-based literature addressing the pharmacological management of chronic pain in older persons is sparse [8]. As a result, algorithms for treatment are mostly generated from study outcomes in younger patients that are extrapolated to older individuals. Since clinical trials in OA knee pain often include patients ranging in age from 40 years to 65 years and older, analyzing treatment outcomes in age-stratified data may provide better clinical insight than extrapolations from younger to older patients.

In clinical studies, duloxetine (a selective, relatively balanced serotonin and norepinephrine reuptake inhibitor) has demonstrated efficacy in four distinct chronic pain conditions: diabetic peripheral neuropathic pain [9,10], fibromyalgia [11,12], chronic low back pain [13,14], and OA knee pain [15,16]. Since the OA knee pain trials included many older patients, we conducted a post hoc analysis of those trials to examine any differential effect of age on the efficacy and safety of duloxetine. In addition, we examined data that might suggest appropriate dosing of duloxetine in this population. In a recently published study, Abou-Raya et al. [17] reported that duloxetine 60 mg/day significantly improved pain as compared with placebo in older patients with OA knee pain, many of whom also had comorbid depression. Our post hoc analysis differs from that study in that the patients in our studies were not depressed, thereby allowing determination of the direct analgesic effect of duloxetine without confounding by improvement in depression. Furthermore, we compared treatment outcomes between older and younger patients, and investigated the utility of increasing the dose in non-responding patients.

## Methods

Data were from two published 13-week, randomized, placebo-controlled studies in male and female outpatients who were at least 40 years of age, and met American College of Rheumatology clinical and radiographic criteria for the diagnosis of OA of the knee [15,16]. In both studies, pain was assessed daily using an ordinal 11-point numerical rating scale (0 to 10) that was recorded in patient diaries. Efficacy assessments were based on the weekly mean of the daily average pain severity from these patient diaries. Pain was also assessed with the Brief Pain Inventory (BPI) [18] (severity range: 0–10) at weeks 4, 7, and 13. All patients provided written informed consent before the commencement of any study procedures.

For both studies, patients were required to have pain for ≥14 days of each month for 3 months prior to study entry, with a mean pain severity ≥4 on daily pain diary ratings during the screening and baseline periods. In addition, patients had to agree to maintain their usual activity level throughout the course of the study. Key exclusion criteria for both studies included having a body mass index >40 kg/m^2^; a diagnosis of inflammatory arthritis or an autoimmune disorder; having received invasive therapies within the prior 3 months, or joint replacement to the knee; being non-ambulatory or needing assistance walking with crutches or walker; any serious medical condition or psychiatric disorder, including major depressive disorder, as identified by the Mini International Neuropsychiatric Interview [19] that could compromise participation in the study.

Patients were randomly assigned 1:1 in double-blind fashion to treatment with duloxetine 60 mg/day or placebo as determined by a computer-generated random sequence using an Interactive Voice Response System (IVRS). All patients randomized to duloxetine were started on 30 mg/day for one week then escalated to 60 mg/day. After 7 weeks, the duloxetine dosing regimen could change. In Study I [15], patients in the duloxetine group were re-randomized via IVRS without regards to change in pain severity to either continue duloxetine 60 mg/day or to have their dose increased directly to 120 mg/day, and were continued on that dose for the remainder of the study. In Study II [16], patients in the duloxetine group who had <30% reduction from baseline in pain severity on the BPI 24-h average pain severity scale, had their dose increased directly to 120 mg/day and continued on that dose for the remainder of the study.

For this post hoc analysis, two patient groups were defined by their age at study entry: older patients were at least 65 years of age, and younger patients were less than 65. For the efficacy and safety analyses comparing duloxetine with placebo within age groups, studies were pooled and doses were pooled. In addition, a subgroup analysis was conducted to determine the efficacy of duloxetine in the “oldest of the old” (>75 years) as compared with the “younger of the old” (65 to <75 years) patients. For comparing the effect of increasing duloxetine to 120 mg in non-responding patients, only data from Study II were evaluated as it most closely reflected clinical practice; whereas re-randomization to a higher dose does not. Treatment arms were defined post hoc as placebo, duloxetine 60 mg/day (patients who remained on 60 mg for the entire study), and duloxetine 60/120 mg/day (patients who received 60 mg for 7 weeks, followed by duloxetine 120 mg for 6 weeks). Safety assessments included spontaneously reported treatment-emergent adverse events (TEAEs), discontinuation rates due to TEAEs, serious adverse events (SAEs), and treatment-emergent abnormal vital signs.

Categorical baseline characteristics were compared between age groups using a logistic regression model with terms for study, treatment, age group and the treatment-by-age group interaction. Quantitative baseline characteristics were compared using an analysis of variance model with similar explanatory terms. Weekly means of change from baseline in the average pain severity ratings from patient diaries were analyzed using a likelihood-based, mixed-effects model repeated measures (MMRM) approach that used all available observations. The model included the fixed categorical effects of site, treatment, week, age group, treatment-by-week interaction, age group-by-week interaction, treatment-by-age group interaction, treatment-by-age-group-by-week interaction, as well as the continuous fixed covariates of baseline score and baseline-by-week interaction.

The analysis of completion rates, discontinuation rates due to adverse events (AEs), and incidence of spontaneously reported TEAEs and SAEs were compared using a logistic regression model with terms for study, treatment, age category, and the treatment-by-age interaction. For comparison among treatment groups and age groups, test results were considered statistically significant if p≤.05; for tests of interaction, results were considered statistically significant if p≤.1. Statistical analyses were performed using SAS, version 9.1 (SAS Institute, Inc, Cary, NC).

## Results

Demographic characteristics and baseline pain severity of patients stratified according to age group and treatment arms are summarized in Table 1. The mean age of the older patients (n=197) was 72 (range, 65 to 87) years; 67.5% were female, and 93.9% were Caucasian. The mean age of the younger patients (n=290) was 56 (range, 40 to 64) years; 73.8% were female; and 89.3% were Caucasian. Most of the patients in each age group were overweight or obese, and the mean BMI at baseline did not differ between treatments or age groups. The duration of OA since diagnosis was significantly longer in the older versus the younger group (p<.001); as was the duration of OA pain since onset (p<.001). NSAID use >14 days/month was significantly less in the older group versus the younger group (p=.03). Differences between age groups in baseline pain diary scores or BPI average pain severity were not significant.

**Table 1:** Baseline demographics and illness characteristics

	**Older**		**Younger**		**Older versus younger**
**Variable**	**Placebo N=94**	**Duloxetine N=103**	**Placebo N=154**	**Duloxetine N=136**	**p values**
Age in years, mean (SD)	72.0 (4.0)	71.4 (4.1)	56.2 (5.7)	56.0 (5.8)	<.001
Female, n (%)	72 (76.6)	61 (59.2)	116 (75.3)	98 (72.1)	.19
Caucasian, n (%)	88 (93.6)	97 (94.2)	136 (88.3)	123 (90.4)	.11
OA duration, years, mean (SD)	8.1 (8.1)	7.9 (8.3)	5.3 (5.5)	5.5 (6.0)	<.001
OA pain, years. mean (SD)	9.4 (8.1)	10.3 (9.1)	7.1 (7.1)	7.2 (7.2)	<.001
BMI (kg/m^2^), mean (SD)	30.0 (4.6)	30.1 (4.1)	30.9 (4.9)	29.9 (5.0)	.45
Pain diary ratings, mean (SD)	6.2 (1.4)	6.0 (1.3)	6.1 (1.2)	6.1 (1.2)	.56
BPI, mean (SD)	6.3 (1.5)	6.0 (1.6)	6.1 (1.4)	6.2 (1.4)	.83
NSAID use, n (%)^a^	39 (41.5)	37 (35.9)	73 (47.4)	68 (50.0)	.03

^a^NSAID use was >14 days/month. Abbreviations: SD, standard deviation; OA, osteoarthritis; BMI, body mass index; BPI, Brief Pain Inventory, NSAID, non-steroidal anti-inflammatory drug.

**Figure 1:**
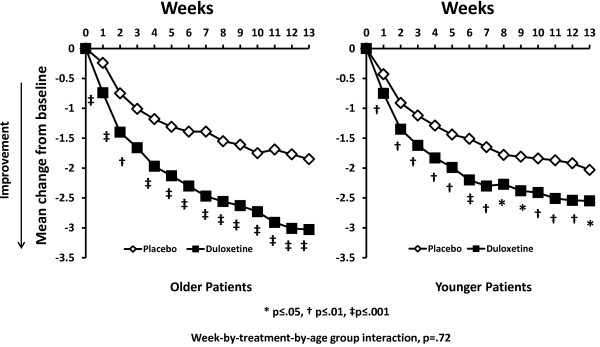
Mean change from baseline in weekly average daily pain diary scores in older and younger patients treated with duloxetine 60/120 mg/day or placebo from pooled study data.

**Figure 2:**
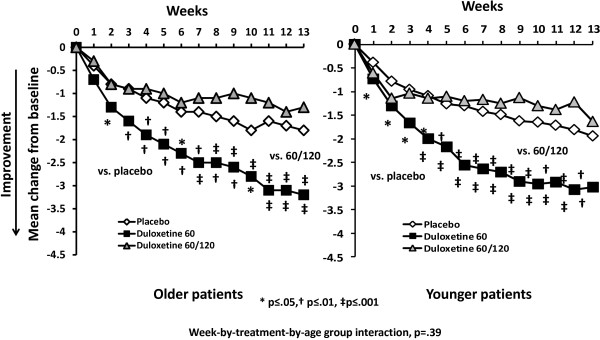
Mean change from baseline in weekly average daily pain diary scores in patients in Study II, who responded to treatment with duloxetine 60 mg/day and remained on that dose; those who were not responding and had their dose increased to 120 mg; and those who received placebo.

Over the course of 13-weeks of treatment, both older and younger patients experienced significantly greater pain reduction each week with duloxetine treatment versus placebo (Figure 1). No statistically significant difference in efficacy between older and younger patients was seen across weeks (age group- by-week-by treatment interaction p-value= 0.72). Among the older patients, there was no significant difference in efficacy between those who were >75 years and those who were <75 years (p=.70). The effect of increasing the dose in non-responding patients was not associated with significant pain reduction versus placebo in subsequent weeks or at endpoint (Figure 2).

Study completion rates did not differ between age groups (p=.69), but significantly more patients treated with placebo versus duloxetine completed the study (older, 84.9% versus 70.9%; younger, 83.1% versus 71.3%; p<.001). There was no significant treatment-by-age group interaction in rates of discontinuation due to adverse events (p=.74), but the rate was significantly greater in patients treated with duloxetine versus placebo (older, 22.3% versus 7.5%; younger, 11.8% versus 4.5%; p<.001), and was significantly greater in older versus younger patients (p=.05). AEs that were reasons for discontinuation in the older group treated with duloxetine included: arthralgia (n=2), asthenia (n=3), nausea (n=4), and one incident for each of these: abnormal dreams, anxiety, bronchitis, diarrhea, drug intolerance, ejaculation disorder, erectile dysfunction, flatulence, headache, hypercreatinemia, insomnia, migraine, rash, and supraventricular tachycardia. In the younger group, patients treated with duloxetine discontinued due to nausea (n=3), and n=1 for each of the following: anxiety, condition aggravated, constipation, dizziness, dyspepsia, fatigue, hemorrhoids, hot flush, insomnia, memory impairment, palpitations, sleep disorder, and somnolence.

TEAEs that occurred with a frequency of ≥5% are summarized in Table 2. Overall, there was no significant treatment-by-age group interaction for the occurrence of at least one TEAE, but within both age groups duloxetine treatment was associated with significantly greater frequency of experiencing at least one TEAE of any type, as well as a greater frequency of patients experiencing constipation and nausea. Of the individual TEAEs, dizziness showed a significant treatment-by-age group interaction with duloxetine treatment, with greater incidence than placebo in younger patients but not in the older patients. Rates of TEAEs that occurred after patients were escalated to duloxetine 120 mg were numerically higher in the younger patients as observed in the older ones (24.4% versus 20%), and these rates were also numerically higher than those observed in patients who remained on the 60 mg dose (14.6% in older and 14.9% in younger patients).

**Table 2:** Treatment-emergent adverse events (TEAE) occurring in at least 5% of patients who received duloxetine

	**Older**		**Younger**		
	**Placebo N=94 n (%)**	**Duloxetine N=103 n (%)**	**Placebo N=154 n (%)**	**Duloxetine N=136 n (%)**	**Treatment-by-Age Group Interaction p values**
Patients with at least one TEAE	40(42.6)	55(53.4)	51 (33.1)	65(47.8)	.66^a^
Constipation	2 (2.1)	11 (10.7)	0 (0)	3 (2.2)	1.00^b^
Diarrhea	2 (2.1)	4 (3.9)	4 (2.6)	7 (5.1)	.93
Dizziness	3 (3.2)	1 (1.0)	1 (0.6)	9 (6.6)	.02
Nausea	3 (3.2)	9 (8.7)	2 (1.3)	11 (8.1)	.40^c^
Somnolence	3 (3.2)	6 (5.8)	1 (0.6)	4 (2.9)	.49
Patients with at least one SAE	2 (2.1)	3 (2.9)	2 (1.3)	1 (0.7)	.57

^a^ treatment effect, p=.005.

^b^ treatment effect, p=.03.

^c^ treatment effect, p=.004.

Abbreviations: *TEAE*, treatment-emergent adverse event; *SAE*, serious adverse event.

The frequency of SAEs did not differ significantly between age or treatment groups and there was no significant treatment-by-age interaction (Table 2). SAEs reported by older patients who received duloxetine (n=1 for each) included: asthma, bronchitis, drug intolerance, rhinitis allergic, and supraventricular tachycardia. Older patients in the placebo group reported atrial fibrillation (n=1), and myocardial infarction (n=1). SAEs in younger patients treated with duloxetine included memory impairment (n=1), younger patients treated with placebo reported dehydration (n=1) and gouty arthritis (n=1). There were no SAEs that occurred in either older or younger patients after escalation to the 120 mg dose of duloxetine. No deaths occurred in either study.

Treatment-emergent vital sign abnormalities are summarized in Table 3. Across age groups and treatment groups, the most common abnormality was orthostatic hypotension. There were no significant differences between age groups, and no statistically significant treatment-by-age-by-visit interactions on treatment-emergent abnormalities in vital signs or weight.

**Table 3:** Treatment-emergent vital sign abnormalities

	**Older**				**Younger**				
	N*	**Placebo n (%)**	N*	**Duloxetine n (%)**	N*	**Placebo n (%)**	N*	**Duloxetine n (%)**	**Treatment-by-Age Group Interaction p values**
PCS Weight gain	92	3 (3.3)	102	0	153	0	128	1 (0.8)	‐‐‐ ^a^
PCS Weight loss	92	0	102	6 (5.9)	153	1 (0.7)	128	2 (1.6)	‐‐‐ ^a^
Sustained hypertension	84	2 (2.4)	95	1 (1.1)	140	2 (1.4)	121	3 (2.5)	.37
Diastolic hypertension	82	0	91	0	135	1 (0.4)	116	0	‐‐‐ ^a^
Systolic hypertension	63	2 (3.2)	78	1 (1.3)	124	2 (1.6)	110	3 (2.7)	.34
Orthostatic hypotension	82	8 (9.8)	98	13 (13.3)	150	8 (5.3)	128	13 (10.2)	.60
Orthostatic tachycardia	93	0	102	0	152	1 (0.7)	136	0	‐‐‐ ^a^

^a^ Treatment-by-age group interaction could not be calculated for diastolic hypertension, PCS weight gain or loss; or for orthostatic tachycardia, because there were 0% values in one or more treatment groups and the model could not fit the data.

Definitions: N*, number of patients at baseline who did not have the abnormality being summarized. PCS (potentially clinically significant) weight gain is ≥7% increase in body weight. PCS (potentially clinically significant) weight loss is ≥5% decrease in body weight. Diastolic hypertension is sitting diastolic blood pressure ≥ 85 mm Hg and increase from baseline of 10 mm Hg for at least 3 consecutive visits. Systolic hypertension is sitting systolic blood pressure ≥ 140 mm Hg and an increase from baseline of 10 mm Hg for at least 3 consecutive visits. Sustained hypertension is having both diastolic and systolic hypertension for at least 3 consecutive visits. Orthostatic hypotension is a decrease of at least 10 mm Hg less than the supine diastolic blood pressure or the standing systolic blood pressure at least 20 mm Hg less than the supine systolic blood pressure. Orthostatic tachycardia is increase of ≥100 beats per minute.

## Discussion

This post hoc analysis did not find a differential effect of age on duloxetine treatment in non-depressed patients with OA knee pain. This is an important finding as it suggests that duloxetine is efficacious in the treatment of OA knee pain regardless of age. The lack of an age effect on pain reduction in this patient population is supported by similar findings in a post hoc age-stratified subgroup analysis of duloxetine in the treatment of diabetic peripheral neuropathic pain [20].

In both older and younger patients, treatment with duloxetine 60 mg/day was associated with significantly greater pain reduction as compared to placebo. However, increasing the dose of duloxetine in older and younger patients, who were not experiencing adequate pain reduction, was not found to provide additional benefit. These findings may be particularly relevant to the treatment of older patients to avoid unnecessary dose escalation.

Overall, duloxetine was generally well tolerated by patients in each age group, considering that rates of completion and discontinuation due to adverse events did not differ significantly or show a tendency toward more drop outs in the older population, and were similar to findings in other studies across indications. Further, adverse events with duloxetine generally did not differ between age groups except that dizziness was associated with greater risk among younger patients relative to older ones. While orthostatic hypotension was the most common treatment-emergent vital sign abnormality reported in each age and treatment group, there was no significant treatment-by-age group interaction.

The interpretation of this post hoc analysis has limitations to be considered. First, neither study was specifically designed to assess safety or efficacy in exclusively older patients. Furthermore, the results of subgroup analyses should generally be interpreted with caution due to concerns about multiple testing and increased likelihood of finding potentially spurious and un-reproducible results [21].

## Conclusions

Duloxetine 60 mg was efficacious for managing OA knee pain in both age groups, but increasing the dose to 120 mg in non-responding patients did not provide additional benefit. There was no consistent signal indicating that the safety of duloxetine might differ significantly between older and younger patients.

## Competing interests

This work was supported by Lilly USA, LLC, Indianapolis, IN, USA.

JLM is on the speaker's bureau and advisory board for Eli Lilly and Company. DR, JA, and MMW are employees and stockholders of Eli Lilly and Company.

## Authors’ contributions

JLM contributed to interpretation of the data and critical review of the manuscript. DR contributed to study design, interpretation of the data, and critical review of the manuscript. JA prepared the manuscript and contributed to interpretation of the data. MMW contributed to study design, interpretation of the data, and critical review of the manuscript. All authors read and approved the final manuscript.

## Pre-publication history

The pre-publication history for this paper can be accessed here:

http://www.biomedcentral.com/1471-2474/14/137/prepub
